# Functionalized NaA nanozeolites labeled with ^224,225^Ra for targeted alpha therapy

**DOI:** 10.1007/s11051-013-2082-7

**Published:** 2013-10-31

**Authors:** Agata Piotrowska, Edyta Leszczuk, Frank Bruchertseifer, Alfred Morgenstern, Aleksander Bilewicz

**Affiliations:** 1 Institute of Nuclear Chemistry and Technology, Dorodna 16, 03-195 Warsaw, Poland; 2 Institute for Transuranium Elements, Joint Research Centre – European Commission, 76125 Karlsruhe, Germany

**Keywords:** Radiotherapy, Nanomedicine, Nanozeolite, Radium radionuclides, Drug delivery

## Abstract

The ^223^Ra, ^224^Ra, and ^225^Ra radioisotopes exhibit very attractive nuclear properties for application in radionuclide therapy. Unfortunately the lack of appropriate bifunctional ligand for radium is the reason why these radionuclides have not found application in receptor-targeted therapy. In the present work, the potential usefulness of the NaA nanozeolite as a carrier for radium radionuclides has been studied. ^224^Ra and ^225^Ra, α-particle emitting radionuclides, have been absorbed in the nanometer-sized NaA zeolite (30–70 nm) through simple ion exchange. ^224,225^Ra-nanozeolites exhibited very high stability in solutions containing physiological salt, EDTA, amino acids, and human serum. To make NaA nanozeolite particles dispersed in water their surface was modified with a silane coupling agent containing poly(ethylene glycol) molecules. This functionalization approach let us covalently attach a biomolecule to the NaA nanozeolite surface.

## Introduction

It has been shown that ionizing radiation emitted by radionuclides is effective in the treatment of various types of cancers (Boyle and Levin 2008; Zoller et al. 2009). To minimize side effects, radiolabeled agents should be used that are selectively taken up by cancer cells, confining the radiation to the tumor, hence minimizing damage to normal cells (Carlsson et al. 2003; Cutler et al. 2013). For this purpose, tumor-selective peptides, monoclonal antibodies (mAbs), or organ-specific proteins are used. Crucial for the success of this approach are bifunctional ligands which can be attached to a protein and can bind the radioactive metal cation rapidly and selectively (Schubiger et al. 1997; Volkert and Hoffman 1998; Neves et al. 2005). The vast majority of radionuclides used in targeted radiotherapy are metals with strong ability to form stable complexes. These metals can be attached to a biomolecule through multidentate cyclic or acyclic ligands. However, there are radionuclides with very attractive nuclear properties, which can be used in nuclear medicine, but cannot be stably bound to biomolecules by bifunctional ligands. To this group belong, for example, radionuclides of radium, which forms very weak complexes.

**Fig. 1:**
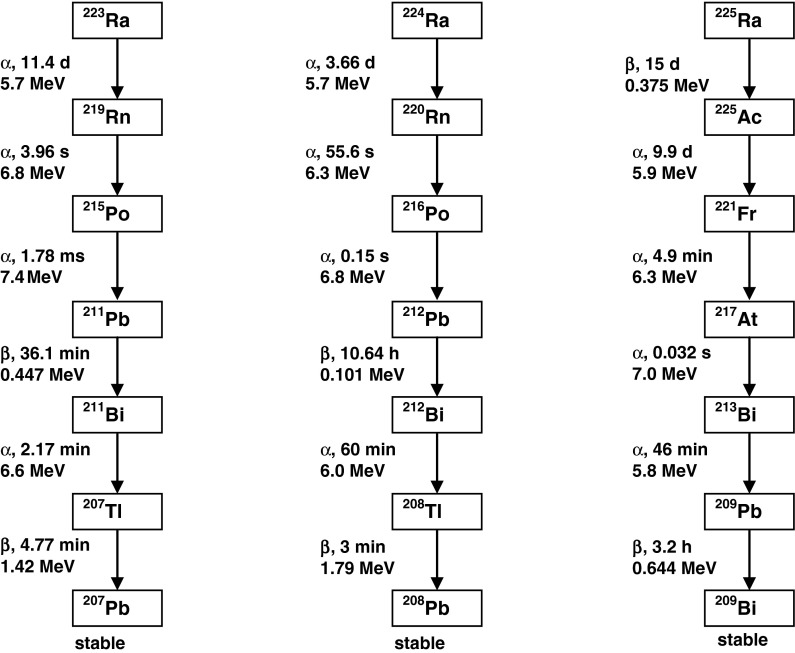
The decay series of radium radionuclides

Radium radioisotopes: ^223^Ra (*t*_1/2_ = 11.43 days), ^224^Ra (*t*_1/2_ = 3.66 days), and ^225^Ra (*t*_1/2_ = 14.8 days) show very attractive nuclear properties for application in radionuclide therapy. These radionuclides decay through a cascade of short-lived *α* and *β* ^−^ particle emitters to stable lead and bismuth, releasing a high total energy of about 28 MeV (Fig. 1). There are four *α* particles emitted in each decay series, thus therapeutically relevant doses can be delivered from low amount of injected activity. The relatively long half-lives give time for preparation, quality control, and shipment of the radiopharmaceuticals. Radium radionuclides can be obtained from their long-living parental radionuclides: ^223^Ra from ^227^Ac (*t*_1/2_ = 21.8 years), ^224^Ra from ^228^Th (*t*_1/2_ = 1.9 years), and ^225^Ra from ^229^Th (*t*_1/2_ = 7,880 years). ^227^Ac can be produced by neutron irradiation of natural radium, ^229^Th by extraction from large stocks of ^233^U, and ^228^Th as the decay product of ^232^U.

The lack of appropriate bifunctional ligand is the reason why radium radionuclides have not found application in receptor-targeted therapy. As already mentioned the Ra^2+^ cation, like other cations of group 2, does not form stable complexes, therefore labeling of biomolecules with ^223,224,225^Ra is a very difficult task. Additionally, a chelating agent is required which could bind radium radionuclide, as well as control the fate of the daughter radionuclides and withstand the immense α-particle recoil energy of 100–200 keV, which is greater than the binding energy in macrocyclic metal ion complexes.

Previous studies on binding ^223^Ra to biomolecules by its complexation with tetraazacarboxylic acids, cryptands, and calixarenes were unsuccessful (Henriksen et al. 2002). Also application of liposomes as carriers for ^225^Ac, ^227^Th, and ^223^ Ra, proposed by Henriksen and co-workers was not brought into practice because of the necessity to carry out many operations with radioactive liposomes (Henriksen et al. 2004).

Nanotechnology can help to overcome these limitations and bring fundamental changes in creating new diagnostic and therapeutic radiopharmaceuticals (Kairem et al. 2008; Ting et al. 2010; Hong et al. 2009). Radionuclides with specific emission properties can be incorporated into nanoparticles (NPs) and used for radionuclide therapy and radio-imaging. NPs are particularly suitable for the delivery of α or β^−^ emitters in radioimmunotherapy. The advantage of NPs is the potential for containing several radioactive atoms within a single carrier. This feature is useful when a target tissue has only a few receptors per cell, which limits the dose that can be delivered (McDevitt et al. 1998).

NPs can deliver radionuclides using either passive or active targeting strategy. The passive targeting accumulation of NPs takes place in non-specific way through enlarged gap junctions in tumor endothelial cells (Carmaliet and Jain 2000). This type of targeting, which enables macromolecules to selectively accumulate in the tumor tissue, is called enhanced permeation and retention (EPR). Unfortunately, local drug deposition is unfeasible for larger tumors with poor vascularization. Target specificity is then achieved through hybrid NPs produced by conjugating NPs with tumor-specific biomolecules, including mAbs, aptamers, peptides, or various receptor-specific substrates (Hamoudeh et al. 2008; Chanda et al. 2010).

The recently investigated nanosystems include, among others, liposomes, silica and silicon NPs (Patel et al. 2010), quantum dots (Patt et al. 2010), noble metals NPs (Chanda et al. 2010; Kucka et al. 2006), dendrimers (Longmire et al. 2008), lanthanum phosphate NPs (Woodward et al. 2011), and polymer-based NPs (Cho et al. 2007). The possibility of combining imaging and therapeutic modalities in a single NP makes them very attractive in personalized medicine (theranostic approach) (Luk et al. 2012).

In the present work, we propose to use nanozeolite whose channels and cavities will be filled with cations of ^223^Ra, ^224^Ra, and ^225^Ra by means of simple ion exchange, without any additional operations.

Zeolites are biocompatible crystalline aluminosilicates composed of tetrahedral structures which build open framework consisting of channels and cages of molecular dimensions. Each tetrahedron containing aluminum formally bears one unit of negative charge, because aluminum atom is connected to four oxygen atoms. To make the crystal electrically neutral metal cations (e.g., Na^+^, K^+^, Ca^2+^) are present in the interstices of the zeolitic framework. We can distinguish many different kinds of zeolite structures with different selectivity and ion-exchange properties (Sherry 1969). The use of nanozeolite with suitable structure should enable stable encapsulation of radium radionuclides inside the framework. In the light of the Eisenman–Sherry theory of cation-exchange selectivity, zeolites exhibit greater or smaller preference for one cation over another (Reichenberg 1966). Taking into account these rules, we can predict that zeolites with high aluminum content, like the A-type nanozeolite, will prefer Ra^2+^ than Na^+^, K^+^, Mg^2+^, and Ca^2+^, i.e., cations with high concentrations in physiological liquids.

Till now, only one paper was devoted to the application of radiolabeled nanozeolite in nuclear medicine. Tsotsalas et al. used nanozeolite L to fill it with γ-emitter ^111^In^3+^, through simple ion exchange (Tsotsalas et al. 2010). The zeolite L has relatively large windows (*r* = 0.35 nm), in comparison with In^3+^ ionic radius (*r*_i_ = 0.080 nm, CN = 6), which results in leakage of ^111^In under physiological conditions. To avoid the leakage, the entrances to the channels were closed by specifically designed silanol stopcocks. The authors concluded that the obtained samples were highly stable in physiological liquids and may enable a large variety of functionalities on the external surface of nanozeolite L. However, the proposed procedure requires difficult post-labeling operations.

The aim of our work was to synthesize the NaA nanozeolite of small dimension (<100 nm), to load it with radium radionuclides, and to study stability of the labeled nanozeolite in various biological liquids.

## Experimental

### Materials

The following chemical reagents were used: Ludox CL-X colloidal silica 45 % suspension in water (Sigma-Aldrich, USA), sodium aluminate (Sigma-Aldrich, Germany), sodium hydroxide (pure p.a., Chempur), 2(methoxy(polyethylenoxy)propyltrimethoxysilane (silane-PEG, abcr GmbH & Co. KG, Germany), ethanol (99,9 %, POCH, Poland), sodium chloride (POCH, Poland), EDTA (POCH, Poland), l-cysteine (Sigma-Aldrich, Japan), tetramethylammonium hydroxide (TMAOH 25 % wt., Sigma-Aldrich, Switzerland), nitric acid (POCH, Poland), hydrochloride acid (POCH, Poland), human serum (obtained from a donor and stored at −20 °C), and distilled water (Millipore).

### Radionuclides

^224^Ra was produced from a generator based on the ^232^U/^228^Th pair (Narbutt and Bilewicz 1998). About 5 MBq of ^232^U was adsorbed in a small column on Teflon grains impregnated with HDEHP. Next, ^224^Ra was eluted with 0.1 M HNO_3_, the eluate from the generator evaporated to dryness and the remaining residue containing radioactivity was re-dissolved in 200 μl of 0.1 M HCl.

^225^Ra was obtained from the ^229^Th source available at the Institute for Transuranium Elements, Karlsruhe, Germany. The source contains 214.5 mg of ^229^Th (*t*_1/2_ = 7,340 years), which makes possible to isolate 1.60 GBq of ^225^Ra every 9 weeks. In order to isolate ^225^Ra in the first step ^225^Ra and ^225^Ac were separated from ^229^Th using ion exchange (Apostolidis et al. 2005), followed by separation of ^225^Ra from ^225^Ac using extraction chromatography (Zielinska et al. 2007). Briefly, thorium batch, consisting of 500 ml of Dowex 1 × 8, anion-exchange resin, was washed 5–6 times using each time 100 ml of 8 M HNO_3_. The combined washing solutions, containing ^225^Ra, ^225^Ac, and 3–5 % of ^229^Th/^232^Th, were evaporated to almost dryness, put into 20 ml of 8 M HNO_3_ and filtered through a preliminary column (1.0 ml volume) to remove organic impurities. Next, the filtrate was loaded into an anion-exchange column (Dowex 1 × 8, 100–200 mesh, column volume 80 ml). ^225^Ra and ^225^Ac were eluted by washing with 250–300 ml of 8 M HNO_3_, and the eluate was evaporated to nearly dryness and put into 10 ml of 4 M HNO_3_ for subsequent radium/actinium separation by extraction chromatography. Subsequently, the ^225^Ra/^225^Ac product from the anion-exchange separation in 4 M HNO_3_ was loaded into a UTEVA/RE-resin column cascade (volume of each equal to 1.6 ml). Under these conditions, residual thorium was extracted on the UTEVA column. Next, ^225^Ra was washed out from the column with no more than 20 ml of 4 M HNO_3_ and evaporated to reduce its volume. Before each experiment, ^225^Ra was purified from its decay products (^225^Ac, ^213^Bi) on a chelating resin. For this aim 1 ml of the solution containing ^225^Ra with its decay products was adsorbed on a small column (*d* = 3 mm, *h* = 20 mm) filled with cation-exchange resin Chelex-100. Next, ^225^Ra was quantitatively eluted with 0.001 M HCl solution.

### Instrumentations

The particle size and morphology of the NaA nanozeolite were determined by scanning electron microscopy (SEM, Zeiss) and transmission electron microscopy (TEM, LEO 912AB). Identification and determination of crystallinity of the nanozeolite were carried out by X-ray diffraction (XRD, D8 Advance) using Vantec detector with Cu Kα radiation (1.54 Å) operated at voltage 40 kV and current 20 mA. The average size and zeta potential (ζ) were measured by dynamic light scattering (DLS, Malvern, UK). Thermal stability of modified and unmodified samples was determined by thermogravimetric analysis (TGA, Q500, TA Instruments, US).

Gamma-spectrometry was carried out using a calibrated intrinsic Ge detector (crystal active volume 100 cm^3^) and PC-based Multichannel Analyzer (MCA, Canberra). The detector had a resolution of 0.8 at 5.9 keV, 1.0 at 123 keV, and 1.9 at 1,332 keV. Radioactivity of ^224^Ra and ^212^Pb was quantified by their 237.75 keV γ-ray, and by the 725.29 keV γ-ray from ^212^Bi. Radioactivity of ^225^Ra was quantified by its 40 keV γ-ray, by the 99.8 keV γ-ray from ^225^Ac, by the 217.86 keV γ-ray from ^221^Fr, and by the 439.8 keV γ-ray from ^213^Bi. The type of the decay product was confirmed by calculation of T_1/2_ from the decay curves.

### Synthesis of NaA nanozeolite

Nanocrystalline NaA zeolite was prepared according to modified procedures described in the papers (Wang et al. 2002; Hu et al. 2009). The aluminosilicate gel composition was

$$\frac{\text{NaOH}}{{{\text{SiO}}_{ 2} }} = 2.21,\quad \frac{\text{TMAOH}}{{{\text{SiO}}_{ 2} }} = 2.21,\quad \frac{{{\text{SiO}}_{ 2} }}{{{\text{Al}}_{ 2} {\text{O}}_{ 3} }}= 3.0,\quad \frac{{{\text{H}}_{ 2} {\text{O}}}}{{{\text{SiO}}_{ 2} }} = 85.79$$

The synthesis procedure was divided into two steps. In the first step, sodium aluminate was added to aqueous sodium hydroxide solution and kept under stirring to complete dissolution. Then the required amount of tetramethylammonium hydroxide (template) was slowly added and the solution stirred continuously for 24 h at room temperature. The second step was the hydrothermal treating. The silica gel was added dropwise to the solution under stirring and the mixture was immediately placed in an oil bath equipped with magnetic stirrer and temperature controller. Hydrothermal crystallization was carried out for 48 h, at 100 °C with rotation rate 750 rpm. The obtained nanocrystals were cooled to room temperature and washed with distilled water by centrifugation at 13,500 rpm (2-16P Spincontrol Universal, Germany) until pH <8. Next, the sample was calcinated at 500 °C for 3 h to remove the template.

### Synthesis of silane-PEG modified NaA nanozoelite

Modified NaA nanozeolite has been synthesized according to the procedure described in the book (Hermanson 2008). The 50 ml of solution containing 4 % water in ethanol (v/v) was prepared and its pH was adjusted to 4.5–5.5 with acetic acid. 2 ml of silane-PEG solution was added dropwise to the acidic water/ethanol solution under stirring at room temperature. The reagents were stirred continuously for 5 min. After that, 50 mg of nanozeolite NaA was added to the solution and stirring was continued for 1 h, at room temperature. The obtained product was washed several times with ethanol to remove excess silane-PEG compound and dried at 110 °C for 30 min.

### Labeling NaA nanozeolite with ^224^Ra and ^225^Ra. Stability studies of ^224/225^Ra-nanozeolites and ^224/225^Ra–nanozeolites-silane-PEG

^224^Ra and ^225^Ra nanozeolites were prepared by exchanging Na^+^ for ^224,225^Ra^2+^. To this end, 100 mg of nanozeolite sample was introduced into the eppendorf tube, suspended in 2 ml of the ^224^RaCl_2_ solution [A ≈ 0.5 MBq], and sonificated in ultrasound bath for 15 min. The eppendorf tube was gently shaken on circular stirrer for 2 h. After that time, the suspension was centrifuged for 10 min at 10,000 rpm (MiniSpin, Eppendorf, Poland). The supernatant was separated from the precipitate and its activity was measured. Afterward, the sample was washed several times with deionized water, then suspended in 1 ml of water and sonificated (Polsonic, Poland). In order to study stability, 100 μl of the ^224^Ra-nanozeolite suspension was incubated in several solutions: saline, PBS buffer, EDTA, l-cysteine, and human serum (at 37 °C) by shaking for 2 h. Then, the suspensions were centrifuged under the same conditions as aforementioned and activities of the supernatants were measured. Afterward, the precipitates were re-suspended in the supernatants and stability studies were extended to 24 h. The same procedure was used for ^225^RaCl_2_ (A ≈ 100 kBq). The stability studies for ^225^Ra were carried out for 4 days. Stability studies of NaA nanozeolites modified with PEG and radiolabeled either with ^224^Ra or ^225^Ra were carried out in exact conditions as described above.

The blank tests with the serum and radionuclide solution, but without nanozeolites were carried out to estimate the percentage of possible binding of free radionuclides (^224,225^Ra^2+^, ^212^Pb^2+^, ^208^Tl^+^, ^212,213^Bi^3+^, ^225^Ac^3+^, ^221^Fr^+^) to proteins present in the serum. After certain time of incubation of blank sample, 1 ml of human serum, combined with 5 μl of ^224,225^RaCl_2_ solution, was centrifuged with the same speed as that used with nanozeolites samples (10,000 rpm) and the activity of the serum taken from the top of centrifuged sample was measured on gamma-spectrometer.

## Results and discussion

**Fig. 2:**
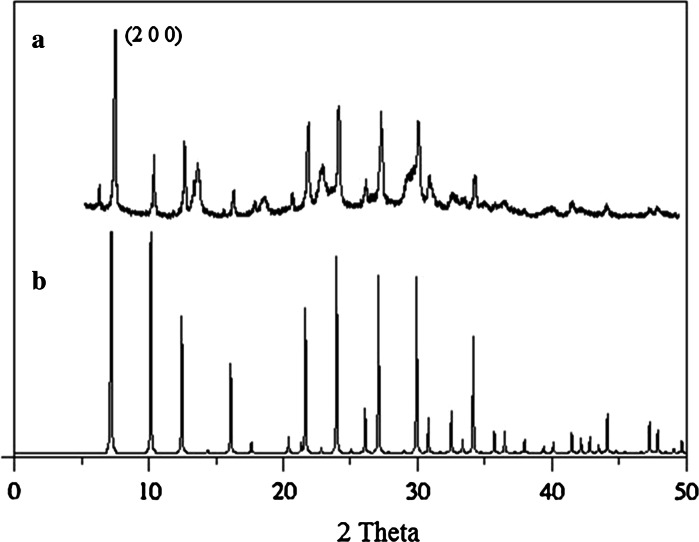
XRD pattern of (*a*) NaA nanozeolite and (*b*) A zeolite

The NaA nanozeolite was successfully prepared by hydrothermal synthesis. Figure 2 shows XRD patterns of the obtained sample. The NaA nanozeolite structure was identified by comparing relative intensities in its XRD powder pattern (Fig. 2a) with those reported in the literature (Fig. 2b) (Gramlich and Meier 1971). The two powder diffraction patterns were found to be in good agreement. The broadened XRD pattern peaks observed in the obtained sample are indicative of small size of the crystalline product.

Crystallinity of the NaA nanozeolite was calculated using Eq. (1) (Cullity 1956):

1 $$\left( {\% \,{\text{C}}} \right) = 100 \times \frac{{I_{\text{hkl}} }}{{I_{\text{b}} + I_{\text{hkl}} }}$$

where *I*_hkl_ is the connected integral XRD peak intensity and *I*_b_ is the integral background intensity for the same plane. The obtained nanozoelite was found to exhibit about 90.9 % of crystallinity.

Scherrer’s formula (Eq. 2) was used to calculate the crystallite dimension (Cullity 1956):

2 $$t = \frac{0.9\,\lambda }{{B{ \cos }\theta_{\text{B}} }}$$

where *B* is the diffraction curve width measured at an intensity equal to half the maximum intensity in radius, *λ* is the X-ray wavelength, and *θ*_B_ is the Bragg angle. The average crystallite size of the NaA nanozeolite is about 43 nm. Both calculations were made with reference to the most significant peak (200).

**Fig. 3:**
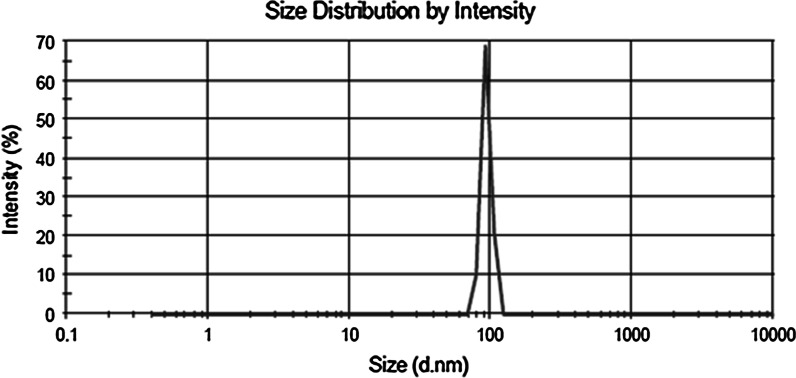
DLS analysis of NaA nanozeolite

**Fig. 4:**
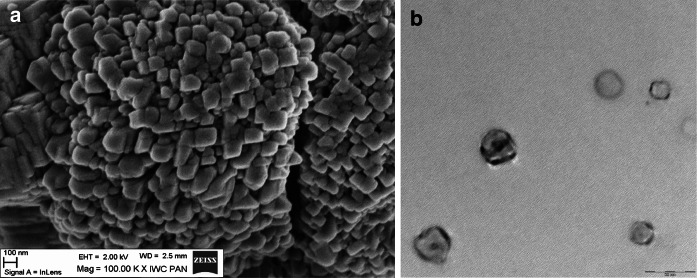
**a** SEM and **b** TEM images of NaA nanozeolite

Size distribution and shape of NaA nanozeolite particles were investigated by DLS (Fig. 3), SEM and TEM (Fig. 4).

It can be seen from the SEM images that the particles with shape similar to parallelepiped, with the size between 50 and 170 nm, were formed. The particles seem to have tendency to aggregate into the size from 200 nm to even 1 μm. However, the DLS analysis shows that the suspended in water NPs are of the size between 40 and 120 nm, which is in good agreement with the SEM results. Disintegration of the agglomerates has been obtained by sonification in an ultrasound bath for 15 min, followed by filtration on syringe filters with 0.22 μm pores (Millipore). To verify the size range of the NPs purified by sonification and filtration the TEM image was made (Fig. 4b). The TEM image shows that the filtration process is crucial for separating the small NPs from agglomerates and for getting a well-dispersed sample.

The obtained sample of the NaA nanozeolite was used for absorption of ^224,225^Ra radionuclides by ion exchange with Na^+^ ions, which are present in the channels and interstices of NPs. The percentage of absorption for both radionuclides was above 99.9 %. The studied nanozeolites exhibited high affinity for both radium isotopes, with the distribution coefficient exceeding 10^4^ cm^3^ g^−1^. The distribution coefficient was calculated as the ratio of the equilibrium activity in 1 g of the solid phase to that in 1 ml of the aqueous phase. Stability of radiolabeled nanozeolites was studied in various solutions containing: physiological salt, complexing agent (EDTA), buffer (PBS), amino acid (l-cysteine), and human serum. In all studied solutions, the leakage of ^224,225^Ra from the nanozeolites was below 0.5 %. In solution only the decay products of radium radionuclides were found. Results of the stability study are shown in Table 1.

**Table 1:** Stability study of the NaA nanozeolite labeled with ^224,225^Ra

Solution	% of liberated radioactivity		
	^224^Ra (1 day of incubation)	^225^Ra (2 h of incubation)	^225^Ra (4 days of incubation)
0.9 % NaCl	0.1 ± 0.1 (^212^Pb)	–	1.1 ± 0.5 (^221^Fr)
	0.7 ± 0.5 (^208^Tl)		3.0 ± 1.2 (^213^Bi)
	1.0 ± 0.3 (^212^Bi)		
0.02 M PBS	0.4 ± 0.1 ^(212^Pb)	–	0.9 ± 0.7 (^221^Fr)
	0.6 ± 0.2 (^208^Tl)		1.8 ± 0.8 (^213^Bi)
	0.6 ± 0.9 (^212^Bi)		
10^−3^ M EDTA	4.8 ± 2.3 (^212^Pb)	0.5 ± 0.02 (^221^Fr, ^213^Bi)	5.5 ± 2.5 (^225^Ac)
	7.8 ± 0.8 (^208^Tl)		5.2 ± 1.4 (^221^Fr)
	9.4 ± 0.6 (^212^Bi)		10 ± 0.8 (^213^Bi)
10^−3^ M cysteine	5.3 ± 3.7 (^212^Pb)	0.4 ± 0.1 (^221^Fr, ^213^Bi)	1.2 ± 0.5 (^225^Ac)
	7.9 ± 5.5 (^208^Tl)		0.8 ± 0.4 (^221^Fr)
	7.5 ± 5.5 (^212^Bi)		13 ± 2.3 (^213^Bi)
Human blood serum	2.6 ± 1.5 (^212^Pb)	–	2.3 ± 1.9 (^225^Ac)
	7.2 ± 0.9 (^208^Tl)		2.7 ± 0.2 (^221^Fr)
	6.2 ± 0.5 (^212^Bi)		7.4 ± 0.8 (^213^Bi)

Table 1 shows the percentage of liberated activity from each daughter radionuclide, determined by its decay characteristic γ-ray peak. The percentage values of each radionuclide refer to the absolute amount of radionuclide in the sample. The NaA nanozeolite labeled with ^224^Ra is very stable in the physiological salt and in PBS buffer solutions. The activities of the samples were near background. The examined sample is less stable in solutions containing either complexing agent (EDTA) or amino acid (l-cysteine). The γ-radioactivity, which was found in the solutions, has been attributed to ^212^Pb, ^208^Tl, ^212^Bi (the decay products of ^224^Ra). When the decay series is taken into consideration, we can assume that due to radioactive equilibrium certain amounts of ^208^Tl and ^212^Bi originate from ^212^Pb decay. But there are also small amounts of these radionuclides, which were eliminated from nanozeolitic structure, due to leaching by aqueous solution. Confirmation of high stability of ^224^Ra-nanozeolite was made after human serum study, which showed that the percentage of released radionuclides was low. The extension of the incubation time to 24 h has brought no changes in the percentage of liberated radioactivity.

^225^Ra has been purified from its decay products before labeling. The purification made possible to obtain NPs labeled only with radium cations, which are strongly bound inside nanozeolite structure. Therefore, there has been no leakage of radioactivity to the solution in 2 days. After that time, the first γ-radioactivity, due to the presence of ^225^Ac, ^221^Fr, and ^213^Bi (the daughter radionuclides of ^225^Ra), has been found. For this reason, stability studies have been enlarged to 4 days. The nanozeolite NaA exhibits also high affinity for the Fr^+^ cation (the biggest alkali metal cations), which results in low leaching of ^221^Fr in the solution. The results were similar to those with ^224^Ra, what means that the assessed values of radionuclide amounts liberated from nanozeolite indicate that there is no leakage of ^225^Ac in physiological salt and in PBS buffer, and the leakage of ^221^Fr and ^213^Bi are negligible. It points out to high stability of radiolabeled NaA nanozeolites in these solutions. Labeled zeolite NPs are unstable in EDTA and cysteine solutions. It should be noted that in EDTA solution whole amount of ^221^Fr originates from ^225^Ac decay, whereas the percentage of ^213^Bi released is twice as big, which shows that this radionuclide was also released from nanozeolites crystals. The same behavior was also observed in cysteine solution. Insignificant amount of radioactivity has been released after incubation in human serum at 37 °C, which points out to high stability of ^225^Ra-nanozeolites. The described blank tests with human serum and free radionuclides have shown that ^224,225^Ra, ^225^Ac, and ^221^Fr radionuclides do not react with proteins present in the serum and that significant amount of ^208^Tl and small amounts of ^212,213^Bi binds to proteins. Thus, the presented percentage values of stability studies with human serum were re-calculated and adequately increased.

**Scheme 1:**
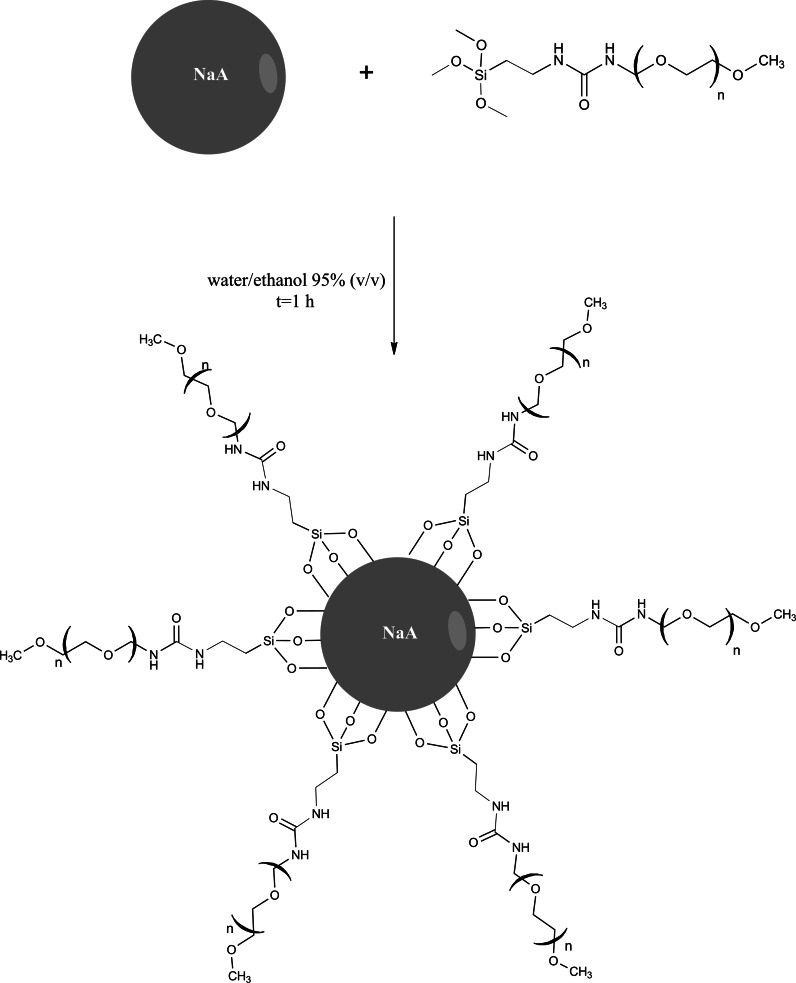
Functionalization of the NaA nanozeolite surface with silane coupling agent (silane-PEG)

**Fig. 5:**
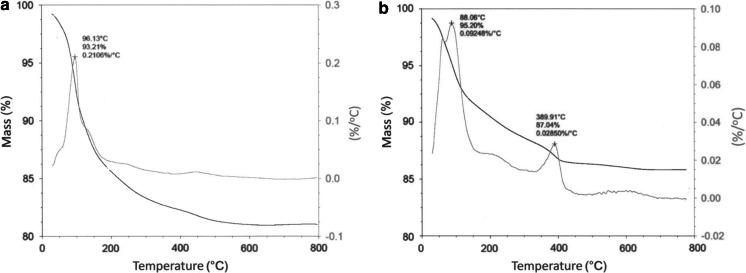
TGA thermograms of **a** unmodified and **b** modified NaA nanozeolite

In the experiments described above unmodified nanozeolites have been used. In order to make the surface of the NaA nanozeolite crystals more hydrophilic and biocompatible, a batch of the nanomaterials has been modified by covalent attachment of PEG molecules. The modification of the NaA nanozeolite surface was fast and simple (Scheme 1). The functionalization of the surface was made by using silane coupling agent with three methoxy groups and PEG molecules. Silane-PEG agent was attached to the surface of NPs by the siloxane bonds formation. The TGA was used to confirm the modification (Fig. 5).

It can be seen on both thermograms that there is a loss of mass (~5 ± 1.5 %) in the range from 80 to 100 °C, which can be attributed to desorption of physically adsorbed water molecules. The consecutive loss of mass is observed between 100 and 800 °C (~8 ± 2 %), what is associated with desorption of water occluded in the interstices and channels of nanozeolite. Additionally, on the modified nanozeolite’s thermogram a loss of mass at about 390 °C (~3 ± 1.5 %) is observed, which can be attributed to degradation of the silane-PEG molecules.

The zeta potential (ξ) was measured in order to give an additional confirmation of the successful modification of NaA nanozeolite surface. We found that the zeta potential has changed from −32.5 ± 1.7 mV for unmodified NPs to −13.4 ± 1.9 mV for modified ones. The zeta potential was measured in phosphate buffer (pH 7.4).

The labeling with ^224,225^Ra and stability studies were also carried out for PEGylated nanozeolite (Table 2). No significant differences were observed between results obtained for functionalized NPs and for those without modification. This indicates that the access to pores and channels of NaA nanozeolite has not been blocked by covering the surface with PEG molecules.

**Table 2:** Stability study of the NaA-silane-PEG NPs labeled with ^224,225^Ra

Solution	% of liberated radioactivity		
	^224^Ra (1 day of incubation)	^225^Ra (2 h of incubation)	^225^Ra (4 days of incubation)
0.9 % NaCl	0.3 ± 0.2 (^212^Pb)	–	1.0 ± 0.4 (^221^Fr)
	1.1 ± 0.8 (^208^Tl)		6.1 ± 3.0 (^213^Bi)
	0.2 ± 0.2 (^212^Bi)		
0.02 M PBS	1.3 ± 0.2 ^(212^Pb)	0.1 ± 0.03 (^225^Ac)	0.8 ± 0.6 (^221^Fr)
	1.5 ± 0.6 (^208^Tl)	0.2 ± 0.09 (^221^Fr)	8.2 ± 1.5 (^213^Bi)
	1.6 ± 0.02 (^212^Bi)	0.2 ± 0.1 (^213^Bi)	
10^−3^ M EDTA	3.5 ± 0.5 (^212^Pb)	0.5 ± 0.2 (^221^Fr)	6.8 ± 3.7 (^225^Ac)
	8.1 ± 0.9 (^208^Tl)	0.4 ± 0.05 (^213^Bi)	11.6 ± 1.7 (^221^Fr)
	11.2 ± 1.5 (^212^Bi)		10.3 ± 1.5 (^213^Bi)
10^−3^ M cysteine	12.0 ± 4.2 (^212^Pb)	0.3 ± 0.02 (^221^Fr)	1.2 ± 0.5 (^225^Ac)
	16.1 ± 6.1 (^208^Tl)	0.4 ± 0.06 (^213^Bi)	0.8 ± 0.4 (^221^Fr)
	17.4 ± 2.0 (^212^Bi)		13 ± 2.3 (^213^Bi)
Human blood serum	6.8 ± 2.3 (^212^Pb)	–	7.2 ± 0.2 (^225^Ac)
	13.9 ± 1.2 (^208^Tl)		5.9 ± 0.4 (^221^Fr)
	7.3 ± 0.7 (^212^Bi)		10.8 ± 2.0 (^213^Bi)

## Conclusions

Our preliminary results have shown that the NaA nanozeolite can be used as a carrier for ^224^Ra and ^225^Ra and for all of their daughter radionuclides. We have also shown that PEG molecules can be covalently attached to the NaA nanozeolite surface using the silanization approach. The modification of the surface makes the NPs stable and dispersed in water, which was confirmed by zeta potential measurement. Additionally, it is worth to notice that ^225^Ra radionuclide is a soft β^−^ emitter, whereas the alpha emission originates from its decay products. Therefore, by formation of radiopharmaceuticals labeled with ^225^Ra, previously separated from all of daughter radionuclides, we can obtain radiobioconjugate with very low β^−^ activity. The significant emission of α-particle occurs from ^225^Ac and its decay products after few days after injection. Thus, ^225^Ra can be used to label macromolecules (for example mAbs) with slow pharmacokinetics and biodistribution time. For example, IgG molecules often reach maximum of accumulation after few days (Henriksen et al. 2004).

In future experiments we intend to use the same procedure of modification, in order to cover the surface with silane coupling agent containing N-hydroxysuccinimide ester and afterward attach targeting vectors like octreotide, substance P or mAbs.
